# Floral Complexity Traits as Predictors of Plant-Bee Interactions in a Mediterranean Pollination Web

**DOI:** 10.3390/plants9111432

**Published:** 2020-10-24

**Authors:** Alon Ornai, Tamar Keasar

**Affiliations:** 1 Evolutionary and Environmental Biology, University of Haifa, Haifa 3498838, Israel; alon.ornai@oranim.ac.il; 2 Biology and the Environment, University of Haifa–Oranim, Tivon 36006, Israel

**Keywords:** pollination network, flower morphology, complexity, phenology, machine learning

## Abstract

Despite intensive research, predicting pairwise species associations in pollination networks remains a challenge. The morphological fit between flowers and pollinators acts as a filter that allows only some species within the network to interact. Previous studies emphasized the depth of floral tubes as a key shape trait that explains the composition of their animal visitors. Yet, additional shape-related parameters, related to the handling difficulty of flowers, may be important as well. We analyzed a dataset of 2288 visits by six bee genera to 53 flowering species in a Mediterranean plant community. We characterized the plant species by five discrete shape parameters, which potentially affect their accessibility to insects: floral shape class, tube depth, symmetry, corolla segmentation and type of reproductive unit. We then trained a random forest machine-learning model to predict visitor identities, based on the shape traits. The model’s predictor variables also included the Julian date on which each bee visit was observed and the year of observation, as proxies for within- and between-season variation in flower and bee abundance. The model attained a classification accuracy of 0.86 (AUC = 0.96). Using only shape parameters as predictors reduced its classification accuracy to 0.76 (AUC = 0.86), while using only the date and year variables resulted in a prediction accuracy of 0.69 (AUC = 0.80). Among the shape-related variables considered, flower shape class was the most important predictor of visitor identity in a logistic regression model. Our study demonstrates the power of machine-learning algorithms for understanding pollination interactions in a species-rich plant community, based on multiple features of flower morphology.

## 1. Introduction

Pollination networks describe community-level pollination interactions and are commonly visualized as bipartite graphs. A large body of research characterizes these graphs and describes their features. Numerous metrics for analyzing pollination networks have been developed, such as connectance, nestedness, modularity and generality [1]. While these measures provide much information on the general organization and robustness of the network, they often fail to predict which pollinators would visit which plant species and why. Forecasting of such specific pairwise interactions has proven to be difficult [2,3] since many species in the networks have several pollination partners [4] and there is a high turnover of species within and across years [5,6].

Information about species traits is increasingly used to interpret the composition of plant communities and to understand the selection pressures that shape them. This effort also includes incorporating knowledge about flower and pollinator traits into pollination network data. The fit between the length of pollinator mouthparts (tongues or bills) and the length of floral tubes has received particular attention in this regard [7,8]. This is because a good fit is needed for efficient bee foraging [9] and effective plant fertilization [10,11]. Yet, additional plant and flower traits were also thought to act as important filters for pollinators, particularly traits related to the shape of flowers or inflorescences. For example, flag- and gullet-shaped flowers restrict the entry of visitors more than disk-shaped flowers [12,13] and flowers with bilateral symmetry are considered more restrictive than radial ones [14]. In this work, we focus on combinations of morphological features that restrict pollinators’ access to flowers, and which have been loosely defined as “floral complexity” in the literature (see [15,16] for reviews). Stefanaki et al. [17], relying on expert evaluations, combined five floral traits that are associated with handling difficulty into a single measure—the “Floral Complexity Index”. The traits that contribute to this index are the flowers’ general shape, tube depth, symmetry, degree of corolla segmentation and type of reproductive unit. Intriguingly, the relative contribution of these shape-related traits as predictors of visitor assemblages have received limited attention so far (but see [18] for an initial analysis). Thus, we do not know sufficiently how the various “complexity” components are perceived and weighted by pollinators. This is the main aim of the present work.

Flowers and pollinators can only interact if they overlap in time [19]. Therefore, the phenology of plants and of insects shapes pollination network metrics, such as specialization, centrality, and modularity [20,21,22]. This was demonstrated by aggregating and analyzing observations of plant–pollinator interactions over different time scales, ranging from a day to an entire flowering season. The resulting network metrics were strongly affected by the temporal scale of analysis. For example, the interactions of both plants and insects became more generalized as the time window considered was increased [1]. Moreover, phenology is a good predictor of two-species interactions in pollination networks, especially when combined with information on floral morphology traits. Plant–pollinator species pairs that are well matched in morphology and phenology form stable and successful associations in pollination networks [23]. Expanding on these findings, we use here the flowering dates of plants in a natural community as an additional species-specific trait. This allowed us to test the importance of phenology-related traits in predicting each flower’s insect visitors, in addition to the multiple features related to floral complexity. We took advantage of the power of machine-learning methods to conduct these analyses. These are algorithms for statistical inference, based on artificial intelligence, that improve automatically from experience. They build a mathematical model based on sample data, known as “training data”, and then use the model to make predictions on a dataset that they had not encountered before (“test data”). In our case, the predictions involve a classification task—namely, correctly forecasting the type of visitor to each flower in the test dataset.

## 2. Results

**Figure 1:**
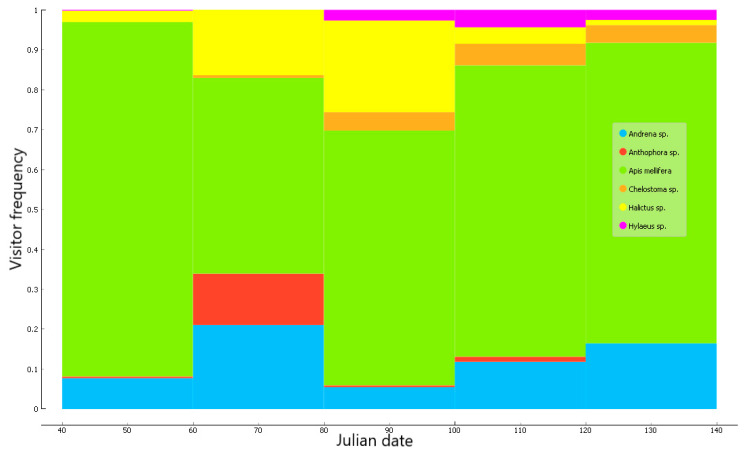
Proportions of all visits due to the six bee genera, observed over the 2015–2016 flowering seasons.

The total number of bee visits did not show a clear trend over the flowering season. Honeybees dominated the bee assemblage and accounted for 67.8% of all flower visits. They were particularly dominant during the beginning of the season (Julian dates 40–60). Large bees of the genus *Anthophora* were active almost exclusively during days 60–80, and *Halictus* spp. peaked during days 80–100. *Hylaeus* and *Chelostoma* foraged in late season (day 80 and onwards), while *Andrena* bees were active throughout the period of observations (Figure 1).

**Figure 2:**
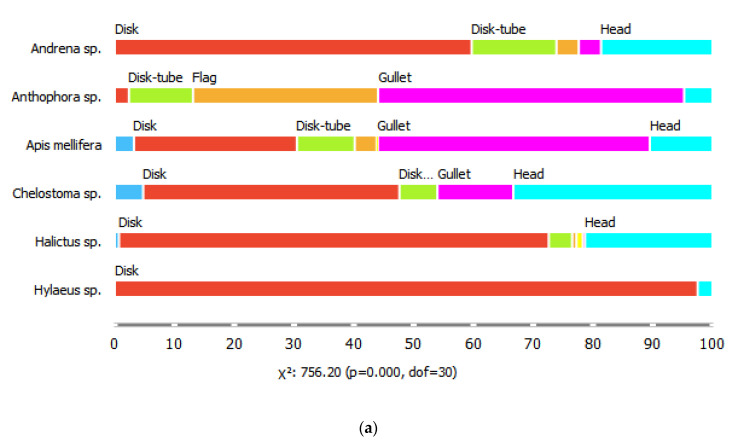

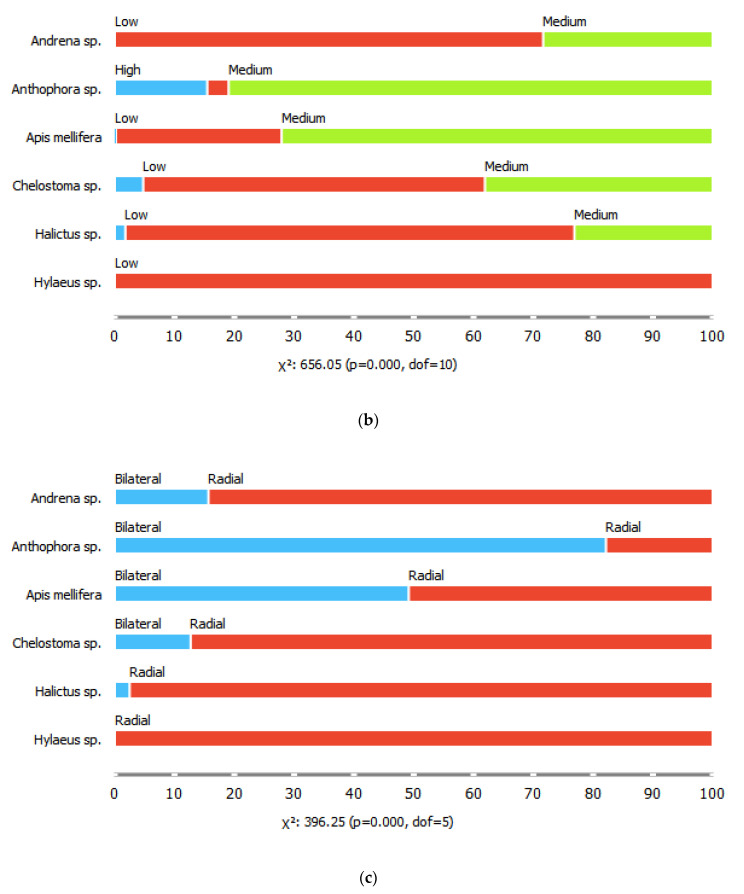

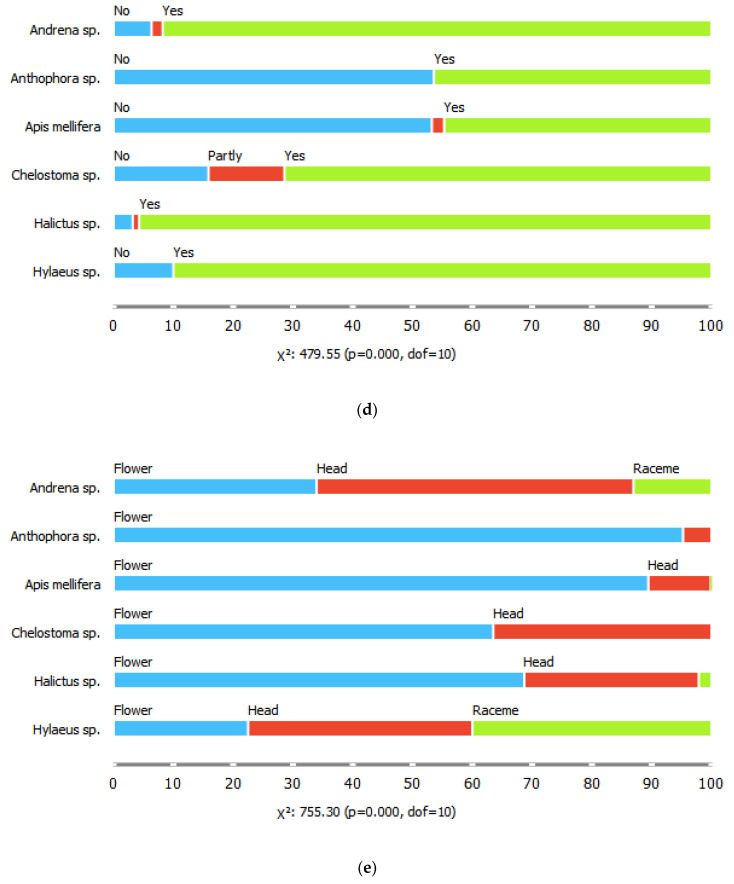
Proportions of (**a**) flower shape classes, (**b**) tube depths, (**c**) symmetry patterns, (**d**) flower segmentation classes, and (**e**) reproductive unit types visited by the six bee genera.

The six common bee genera also differed in their proportions of visits to flowers of different shapes, for all shape parameters considered (chi-square tests, *p* < 0.001). For example, bees of the genus *Hyleaus* visited mostly shallow, disk-shaped flowers, with radial symmetry and segmented corollas. They also visited racemose inflorescences more often than the other genera. Honeybees visited the highest diversity of flower shapes, while *Anthophora* spp. were the most likely to visit gullet-shaped, medium depth, single flowers with bilateral symmetry (Figure 2).

We next used random forest models to predict which bee genus would visit each flower from the dataset of field observations. Given the year and Julian date on which a flower was observed and its five shape-related traits, the model’s classification accuracy (overall proportion of correctly predicted visits) was 0.86. Its AUC score (area under the curve that plots the model’s true-positive vs. false-positive prediction rates) was 0.96. Because of the class imbalance in the data (the class “Honeybee” being much more common than the other bee genera), a high classification accuracy may simply result from overprediction of the most common class by the statistical model. To estimate the effect of the class imbalance on the model’s performance, we artificially reduced the imbalance through two manipulations of the data: randomly leaving out all but 100 visits by honeybees, and combining all non-honeybee visitors into a single class. The model showed only a slightly better performance on the modified datasets than on the original imbalanced data (model with 100 honeybee visits—accuracy: 0.86, AUC: 0.97; model with two visitor classes—accuracy: 0.91, AUC: 0.97). To understand how the model’s classification performance could be further improved, we looked at its types and frequencies of prediction errors (Table 1). The most common errors were mispredictions of visits by *Anthophora* as visits by honeybees, and visits by *Hylaeus* and *Chelostoma* as visits by *Andrena* (Table 1).

Removal of the sampling date and year from the model, using only the shape variables as predictors, reduced its prediction accuracy to 0.76 and the AUC score to 0.86. Visits by *Anthophora*, *Chelostoma* and *Halictus* were particularly poorly predicted (Table 2). Removal of shape traits (i.e., predicting visitors from sampling date and year alone) resulted in an even lower accuracy (0.69) and AUC (0.80). In particular, no visits by *Anthophora* and *Hylaeus* were predicted (Table 3). Both partial models predicted honeybees as visitors far more often than their occurrence in the actual field data (Table 2 and Table 3).

Finally, we ran a logistic regression model on the dataset of flower visits. This model incorporated both shape-related and phenology-related parameters as predictor variables and considered the presence/absence of a visit by each of the six bee genera as a binary response variable. This model correctly predicted only 0.74 of the bee visitors (AUC = 0.88), but provides some insight regarding the relative importance of the different predictors. Shape class was the most important variable in the complete regression model and also in predicting visits of four of the bee genera (*Andrena*, *Anthophora*, *Apis* and *Hyleaus*). For *Chelostoma* and *Halictus*, on the other hand, floral tube depth best predicted whether they would visit a flower or not. The phenology-related variables were generally less important predictors of visitor identity than the shape-related variables (Table 4).

## 3. Discussion

Flowers that restrict pollinator access to their food rewards are considered complex, but how do animal visitors actually perceive and evaluate the different components of “complexity”? Our main aim was to address this question, by combining information on complexity-related floral features with observations of bee visits in a natural species-rich plant community. The use of statistical models based on machine learning is a novel aspect of our analysis.

Floral shape class was the most important predictor of visits for four of the six bee genera in our dataset, and the importance ranking of the shape-related traits varied among bee genera (Table 4). Flower tube depth was the most important trait for predicting visits by honeybees (visiting mainly flowers of medium depth) and *Hylaeus* (visiting only shallow flowers, Figure 2). The importance of the type of reproductive unit varied between bee genera, but it was almost as important as shape class (importance scores: 0.053 and 0.054, respectively) in the statistical model that incorporated all bees (Table 4). Thus, different bees seem to respond to different combinations of shape-related flower traits. This is in line with previous studies, which combined information on flower shape and color traits to predict flowers’ visitor composition at the order level [18,24]. Our results go a step further, and suggest that different combinations of flower morphological traits also attract specific bee genera.

Flower tube length is often suggested as a key trait for understanding pollination interactions. For example, the number of visitor species to a flower species could be predicted from its tube length, width, and its abundance in a Mediterranean plant community [7]. Furthermore, the removal of either all short-tubed or all long-tubed flowers from experimental plots resulted in consistent changes to the visiting insect community [25]. Interestingly, flower tube length was not the most important floral feature for the majority of the bee genera in the present work. This supports the notion that considering multiple flower traits provides better insights on the organization of pollination webs than any single variable. In fact, additional variables such as flower color, corolla area and plant height [20] affect the diversity of plant visitors and are likely to improve predictions of pollinator composition even further. Future analyses may also benefit from describing flower traits, whenever possible, as continuous variables (e.g., tube depth), rather than classifying them into discrete classes.

For two species to interact in a pollination network, their phenologies obviously need to overlap. Additionally, the requirement for morphological fit between flowers and their animal visitors allows only a subset of all possible two-species interactions in the network to occur. Our random forest statistical model, informed by both shape- and phenology-related variables, had a higher prediction accuracy than models that used only one shape or phenology as explanatory variables. Thus, our analysis confirms the importance of both the morphological fit and the temporal matching for predicting flower visitors in a Mediterranean pollination network. Previous studies, conducted in other floristic regions and analyzed with different statistical approaches, reached similar conclusions. Estimates of the ranges of floral trait combinations that are exploited by different pollinator species in a temperate plant community showed strong effects of flower phenology and morphology [20]. Similarly, structural equation modeling of an Argentinian pollination network revealed that phenological and morphological matching between flowers and insects generate stable pollination associations [23].

All three random forest models overpredicted visits by honeybees, the most common pollinator in the dataset. This bias was particularly strong in the two reduced models, where the predictions were based on a smaller number of explanatory variables than in the full model (Table 1, Table 2 and Table 3). Lacking sufficient information on flower traits, the reduced statistical models often resorted to predicting the most common visitor. To reliably predict the rare visitors, a larger dataset is probably needed. Similarly, in the full model (Table 1), the more common genera (*Andrena*, *Apis* and *Halictus*) were predicted with a higher accuracy than the rare ones (*Anthophora*, *Chelostoma* and *Hylaeus*). Interestingly, the errors in predictions often involve bees of similar body sizes (Table 5): *Anthophora* were most often misclassified as the similar-sized *Apis*, and *Chelostoma* were often wrongly predicted to be the similar-sized *Andrena*. Further errors in classification were between the two smallest genera, *Hylaeus* and *Andrena*. Since information about bee morphology was not included in the models, these errors seem to indicate that similarly sized bees choose flowers with similar traits [26]. Adding bee traits (such as body size, voltinism, sociality and nesting habit) into the models is an obvious direction for future work. Such modeling will reveal whether, indeed, erroneous predictions of flower-visitor matching are due to trait overlaps between bees.

Previous studies from Mediterranean ecosystems found that plants with simple flower shapes increase in abundance towards the end of the flowering season, as do small-bodied pollinator species (reviewed in [16]). Similarly, in our dataset, the largest wild bee (*Anthophora*) was active in the early season while the smallest bee (*Hylaeus*) foraged towards its end (Figure 1). This may explain why the phenological variables are relatively more important as predictors of *Anthophora* and *Hylaeus* than for other bee genera (Table 4).

Machine-learning algorithms provide an intuitive way to integrate species-level traits with information on the structure and function of ecological interaction networks. Models that incorporate functional traits have been advocated for predicting the effects of climate change, biological invasions and other anthropogenic impacts on ecological communities [27,28]. Mediterranean pollination networks are rich, complex and often exposed to intensive human activity. We view the present project as a step towards understanding the factors that shape these networks.

## 4. Materials and Methods

### 4.1. The Plant-Visitor Dataset

We analyzed data from 2015–2016, collected during an experiment on forest management practices for fire prevention [29]. The study was conducted in the Mt. Carmel National Park, Israel. The area has a typical Mediterranean climate with long, hot, and dry summers (average daily summer temperatures during May–August are 26.2 ± 1.0 °C, with no significant precipitation) and short cool winters (average daily winter temperatures during November–January are 18.6 ± 1.0 °C). The average annual rainfall is 500 mm, of which 99% occurs during October–April.

The reserve comprises 150 km^2^ of Aleppo pine (*Pinus halepensis* Mill.) woodland in a mosaic of areas that differ in the age of their post-fire regenerating garrigue vegetation. *Pinus halepensis* and *Quercus calliprinos* Webb. are the dominant trees accompanied by multi-stemmed trees, shrubs and dwarf-shrubs (e.g., *Pistacia lentiscus* L., *Cistus salvifolius* L., *Salvia fruticosa* Mill.). Numerous species of herbaceous annuals flower in the reserve, mostly between February and May [30,31].

In total, 32 quadratic plots of 20 × 20 m were selected for the study. Sixteen of them had been burned in a large forest fire in 2010, while the remaining plots had not been burned for at least 20 years. Eight of the burned plots and eight of the unburnt plots were subject to mild sheep grazing (100 h of grazing/month during January–August 2015), and the rest of the plots were fenced and ungrazed. Previous analyses showed that the management treatment and the time since the last fire had significant effects on the composition of flowering plants in the plots, but not on the abundance and composition of insect visitors [29]. This allowed us to exclude fire history and grazing management as factors that influence the identity of flower-visiting bees. Data from all plots were therefore pooled for the present analyses, regardless of past fires and management treatment. Each plot was sampled twice during February–May 2015, and once again between February and April 2016. A sample consisted of a 15-min transect walk along the two diagonals of each plot (56 m). Bee visits to flowers within 1 m on either side of the transects were recorded. Plants were identified to species, and bees were identified to genus, mostly without capture because of conservation concerns. The accuracy of the bee identification is estimated at 94%, based on a subsample of the bees that were identified on the fly, captured and verified by consultation with experts. The transect walks were conducted between 800 and 1400 h, on days with no rain and at temperatures above 18.0 °C. The order by which the plots were sampled was randomized within each of the three sampling rounds. To estimate the abundance of flowers in the plots, we counted the flowers of each blooming species in 1 × 1 m sampling quadrants, placed at 1-m intervals along the plots’ diagonals. Only quadrants with >50% exposed soil area, which were suitable for plant growth, were used for flower counts. The number of observation quadrants therefore varied among plots, and ranged from 8 to 17 (mean ± SE: 12.94 ± 1.66). The number of flowers of each species, summed over all sampling quadrants, is reported in Supplementary Materials Table S1.

### 4.2. Flower Shape and Phenology Traits

We considered all the visited flower species in our analysis, regardless of their abundance and the frequency of bee visits. Following [17], we tabulated five shape-related discrete traits for each plant species: (a) flower shape class: bell, disk, disk-tube, flag, funnel, gullet or head (see Figures S1–S7 for examples); (b) depth (length of floral tube): high (>14 mm), medium (4–13 mm) or low (<3 mm); (c) symmetry: radial or bilateral; (d) corolla segmentation: segmented, partly segmented or fused; (e) reproductive unit: single flower, head (dish-shaped inflorescence) or raceme (umbrella-shaped inflorescence). We also tabulated the date on which each visit was observed and the observation year (2015 or 2016). We used these variables as proxies for the seasonal and annual variation in the composition of the community.

### 4.3. Bees

Bee genera with fewer than 30 visits were excluded from the dataset. This left us with six genera: *Hyaelus*, *Chelostoma*, *Halictus*, *Andrena*, *Apis* and *Anthophora*. We measured the proboscis length and thorax width of 10–15 individuals of each genus (82 bees altogether) that had been captured at the study site, as an indication of their relative body sizes (Table 5).

### 4.4. Data Analysis

The resulting dataset contained six bee genera, 53 plant species and 2288 visit records. We used chi-square tests to examine whether the different categories of the shape-related flower traits were visited proportionally to their frequencies, by each of the six bee genera. All remaining analyses were conducted with the Orange Data Mining (version 3.26) software (Bioinformatics Lab at University of Ljubljana, Ljubljana, Slovenia) [32], using the software’s default settings (Table S2). The raw data and the script used for the analyses are provided as Supplementary files (Table S3 and Code S1).

#### 4.4.1. Predicting the Visitors

We used random forest machine-learning models to predict the bee identity for each visit. The random forest algorithm is a classification algorithm that consists of several (ten, in our case) decision trees. Each of these decision trees had an identical task: to split its input dataset of flower visits into six subsets, each corresponding to a different genus of visiting bees. This was carried out through a series of choices that form the decision tree. One such sequence of choices might start, for example, by first dividing the dataset by floral symmetry, then by flower depth. Another possible sequence might use flower depth as its first decision point, thus giving it greater weight in the classification process. The output of each decision-tree algorithm is the sequence of choices that generates an optimal classification of the data into subsets.

In our random forest model, each of the ten decision trees received inputs of 2288 flower visits, which were obtained by random sampling with replacement (bagging) of the original dataset. Additionally, in each tree only two flower shape/phenology traits were randomly drawn for consideration at each decision point, from which the best attribute for the split was selected. Thus, both the input data and the options for decision-making differed between the ten trees. This resulted in an uncorrelated forest of trees with different outputs. The final model was based on the majority vote from the individually developed trees in the forest. We chose to use random forest models because they performed well in a comparison of algorithms aimed to predict species interactions in ecological networks [28]. The model was trained on 70% of the dataset (1602 randomly selected observations) and tested on the remaining 686 records of visits. The full model included the five shape-related traits and the two phenology-related traits as explanatory variables. Visitor identity was defined as the target variable to be predicted. We also ran two reduced models: one included only shape-related traits and the other only phenology-related traits as explanatory variables. We recorded the classification accuracy (proportion of correctly predicted visitors) and the AUC score (area under the curve that plots a model’s true-positive vs. false-positive prediction rates), and generated a confusion matrix for each model. AUC scores are considered more accurate measures of model performance than classification accuracy, as the AUC is robust regarding unbalanced class sizes (which is the case for our dataset). Classification accuracy, on the other hand, has a more intuitive interpretation than AUC. We therefore report both performance criteria.

#### 4.4.2. Ranking the Predictors

Random forest models are powerful in terms of predictive power, but the relative contribution of the various explanatory variables to the models’ predictions is often hard to interpret. This is because the algorithm’s prediction combines the output of multiple decision trees, which constitute the forest. Because of this complexity, random forest models are frequently considered to be “black boxes”. To gain better insight into the importance of the shape- and phenology-related variables for predicting visitor identity, we also analyzed our dataset using a logistic regression model. This model aimed to predict, for each bee genus, whether it would visit each flower in the observation dataset or not, based on the flower’s shape and phenology traits. Thus, the model’s response variable is binary, and it considers each bee genus separately as its dependent variable. Dependence among the predictors (multi-collinearity) interferes with this type of models. Therefore, following [18], we excluded flower symmetry from the logistic regression because it is highly correlated with flower shape class: all flag- and gullet-shaped flowers are bilaterally symmetrical, while the remaining shape classes have radial symmetry. We used the same training and test datasets as with the random forest model. Namely, we trained the logistic regression model on a random selection of 70% of the observed visits in the dataset, and tested its performance on the remaining 30% of observations. We used the Gini reduction index as a metric of predictor importance in the logistic regression model. Variables that accurately predict the presence/absence of a bee visitor, for a large number of observations, are assigned high Gini reduction scores.

## Figures and Tables

**Table 1:** Confusion matrices summarizing the percentages of visitors correctly identified by the random forest models (shaded diagonals), and the frequencies of misidentifications. Table 1 summarizes a model that included five shape-related and two phenology-related explanatory variables.

Predicted								
		*Andrena* spp.	*Anthophora* spp.	*Apis mellifera*	*Chelostoma* spp.	*Halictus* spp.	*Hylaeus* spp.	∑
**Actual**	*Andrena* spp.	71.6%	0.0%	21.0%	0.0%	7.4%	0.0%	81
	*Anthophora* spp.	4.8%	52.4%	42.9%	0.0%	0.0%	0.0%	21
	*Apis mellifera*	0.4%	0.2%	97.6%	0.2%	1.5%	0.0%	460
	*Chelostoma* spp.	33.3%	0.0%	19.0%	42.9%	4.8%	0.0%	21
	*Halictus* spp.	3.4%	0.0%	24.7%	0.0%	71.9%	0.0%	89
	*Hylaeus* spp.	35.7%	0.0%	21.4%	0.0%	0.0%	42.9%	14
	∑	76	12	504	10	78	6	686

**Table 2:** Confusion matrices summarizing the percentages of visitors correctly identified by the random forest models (shaded diagonals), and the frequencies of misidentifications. Table 2 and Table 3, respectively, summarize models with only shape-related or only phenology-related explanatory variables.

Predicted								
		*Andrena* spp.	*Anthophora* spp.	*Apis mellifera*	*Chelostoma* spp.	*Halictus* spp.	*Hylaeus* spp.	∑
**Actual**	*Andrena* spp.	59.3%	0.0%	34.6%	0.0%	2.5%	3.7%	81
	*Anthophora* spp.	4.8%	9.5%	85.7%	0.0%	0.0%	0.0%	21
	*Apis mellifera*	0.4%	0.0%	98.3%	0.0%	1.3%	0.0%	460
	*Chelostoma* spp.	42.9%	0.0%	47.6%	4.8%	4.8%	0.0%	21
	*Halictus* spp.	12.4%	0.0%	70.8%	0.0%	16.9%	0.0%	89
	*Hylaeus* spp.	50.0%	0.0%	14.3%	0.0%	0.0%	35.7%	14
	∑	78	2	573	1	24	8	686

**Table 3:** Confusion matrices summarizing the percentages of visitors correctly identified by the random forest models (shaded diagonals), and the frequencies of misidentifications. Table 2 and Table 3, respectively, summarize models with only shape-related or only phenology-related explanatory variables.

Predicted								
		*Andrena* spp.	*Anthophora* spp.	*Apis mellifera*	*Chelostoma* spp.	*Halictus* spp.	*Hylaeus* spp.	∑
**Actual**	*Andrena* spp.	22.2%	0.0%	69.1%	1.2%	7.4%	0.0%	81
	*Anthophora* spp.	42.9%	0.0%	57.1%	0.0%	0.0%	0.0%	21
	*Apis mellifera*	3.7%	0.0%	89.3%	0.9%	6.1%	0.0%	460
	*Chelostoma* spp.	0.0%	0.0%	66.7%	23.8%	9.5%	0.0%	21
	*Halictus* spp.	1.1%	0.0%	53.9%	0.0%	44.9%	0.0%	89
	*Hylaeus* spp.	14.3%	0.0%	57.1%	0.0%	28.6%	0.0%	14
	∑	47	0	549	10	80	0	686

**Table 4:** Gini decrease importance estimates for the factors in the logistic regression. Symmetry was deleted from the model because it is strongly correlated with shape class. The variable of highest importance in each column is indicated in red.

Factor	Full Model	*Andrena*	*Anthophora* Sp.	*Apis mellifera*	*Chelostoma* Sp.	*Halictus* Sp.	*Hylaeus* Sp.
Shape class	0.054	10.109	11.374	6.762	21.001	3.507	0.854
Reproductive Unit	0.053	2.427	6.719	4.282	7.923	2.567	1.578
Depth	0.046	8.849	7.121	7.841	12.841	2.796	10.138
Corolla Segmentation	0.045	2.745	3.976	6.767	2.623	2.522	1.258
Julian date	0.031	0.227	0.922	0.191	0.616	0.418	0.814
Year	0.002	0.044	0.878	0.073	0.571	0.280	0.848

**Table 5:** Numbers of flower visits observed by the six bee genera during the 2015–2016 field seasons. Bees are tabulated from small to large, according to their proboscis length.

Bee Genus	Number of Observations in the Visitation Dataset	Mean ± SE Proboscis Length (mm)	Mean ± SE Thorax Width (mm)
*Hyaelus*	40	0.481 ± 0.041	1.523 ± 0.058
*Andrena*	268	0.997 ± 0.140	1.884 ± 0.072
*Halictus*	281	1.861 ± 0.251	2.213 ± 0.110
*Chelostoma*	63	2.002 ± 0.340	1.823 ± 0.103
*Anthophora*	84	3.868 ± 0.943	4.306 ± 0.143
*Apis*	1552	4.006 ± 0.154	4.376 ± 0.057

## Supplementary Materials

The following are available online at https://www.mdpi.com/2223-7747/9/11/1432/s1, Figures S1—S7: Example images of the flower shape classes, photographs by Shai Shafir. Figure S1—bell, Figure S2—disk, Figure S3—disk-tube, Figure S4—flag, Figure S5—funnel, Figure S6—Gullet, Figure S7—Head. Table S1: Estimates of flower abundance, per plant species and sampling session. Table S2: Parameter values for the random forest and logistic regression models. Table S3: Raw data. Code S1: The Orange script used for statistical analyses.
