# Supplementary material for: Floral Complexity Traits as Predictors of Plant-Bee Interactions in a Mediterranean Pollination Web
Source: Plants (Basel). 2020 Oct 24;9(11):1432. doi: 10.3390/plants9111432 (PMC7694153; doi:10.3390/plants9111432)
Supplement: Supplementary file 1 [file plants-09-01432-s001.zip › Supplementary for paper/Table S2 Model parameters.docx]

Table S3: Parameter values for the machine learning models

| Model | Parameter | Value (software’s default setting) |
| --- | --- | --- |
| Random forest | Number of trees | 10 |
|  | Number of attributes considered at each split | 2 |
|  | Minimal subset size | 4 |
| Logistic regression | Regularization type | Lasso |
|  | Regularization strength | Weak (1000) |
