# Supplementary figures and images for: Floral Complexity Traits as Predictors of Plant-Bee Interactions in a Mediterranean Pollination Web
Source: Plants (Basel). 2020 Oct 24;9(11):1432. doi: 10.3390/plants9111432 (PMC7694153; doi:10.3390/plants9111432)

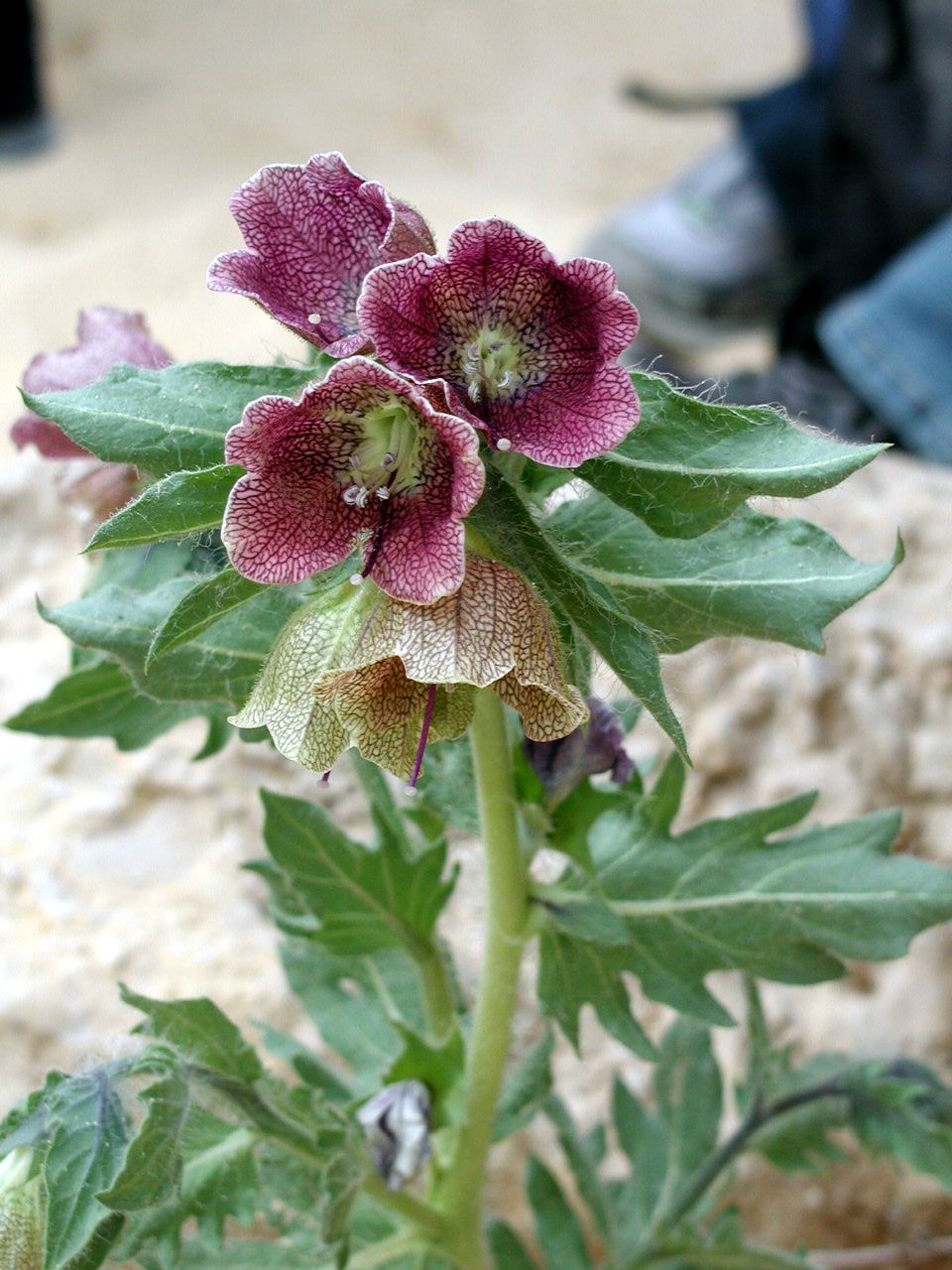

Supplement: Supplementary file 1 [file plants-09-01432-s001.zip › Supplementary for paper/Fig. S1 Bell.jpg]

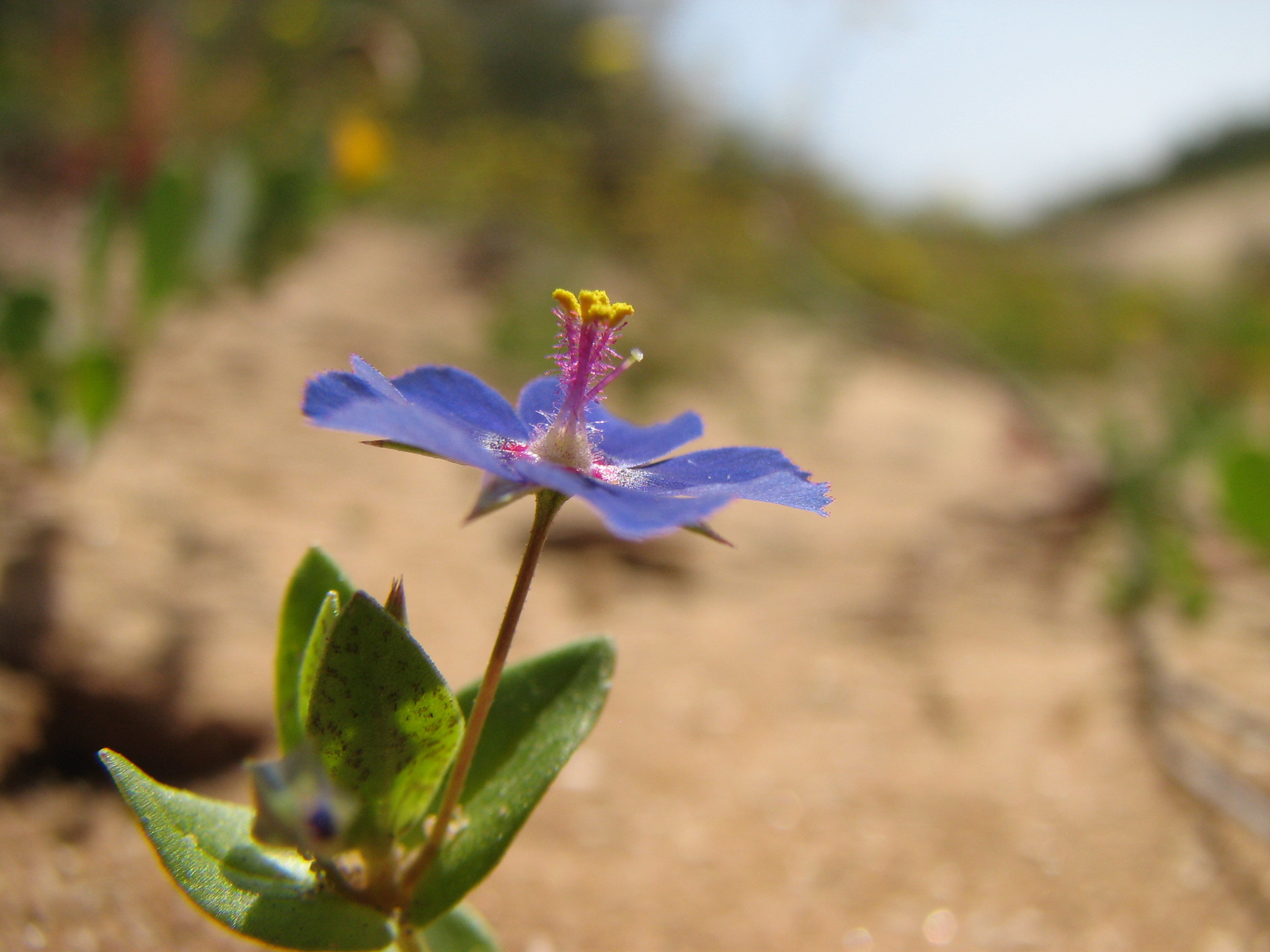

Supplement: Supplementary file 1 [file plants-09-01432-s001.zip › Supplementary for paper/Fig. S2 Disk.jpg]

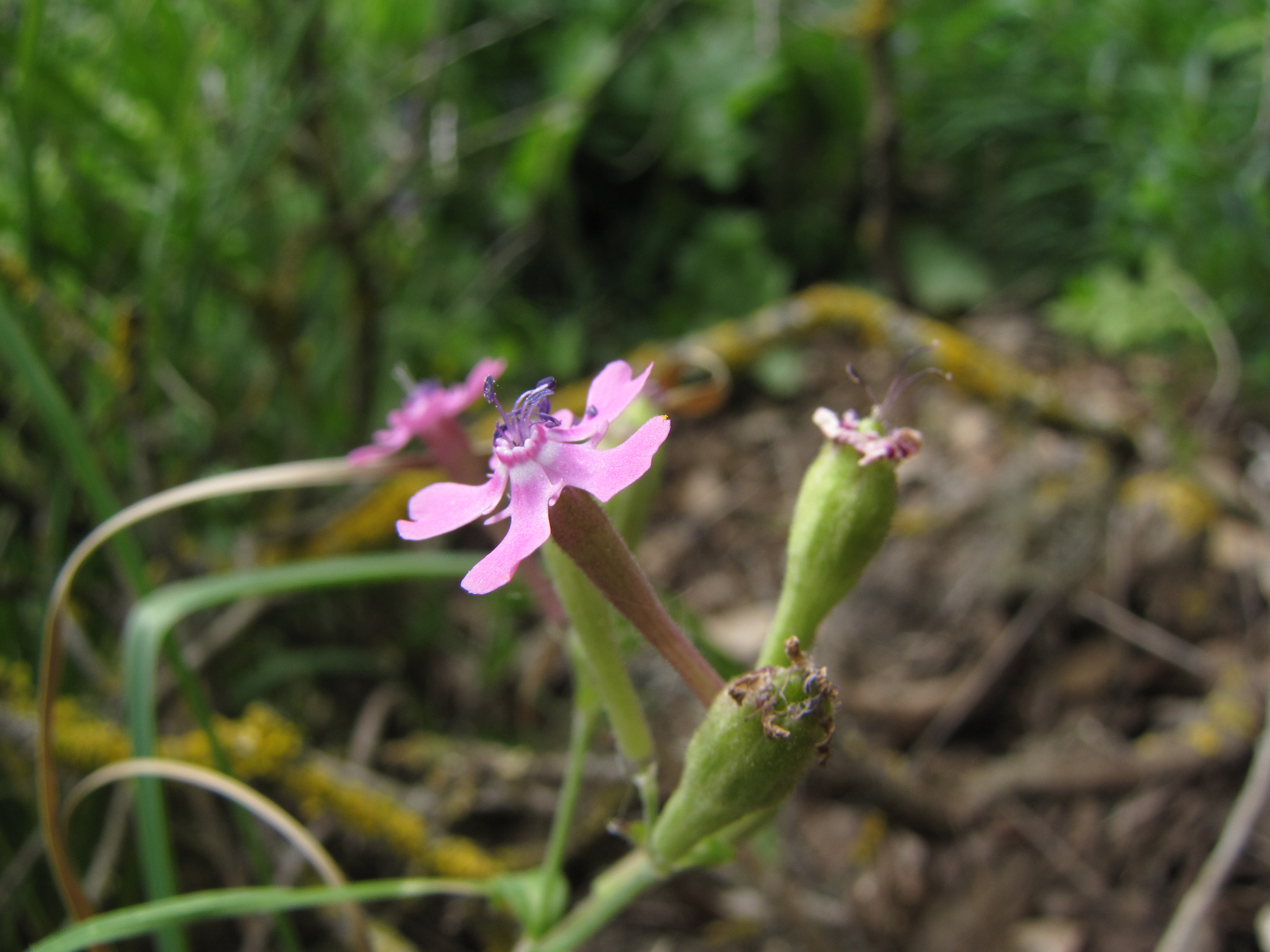

Supplement: Supplementary file 1 [file plants-09-01432-s001.zip › Supplementary for paper/Fig. S3 Disk-tube.jpg]

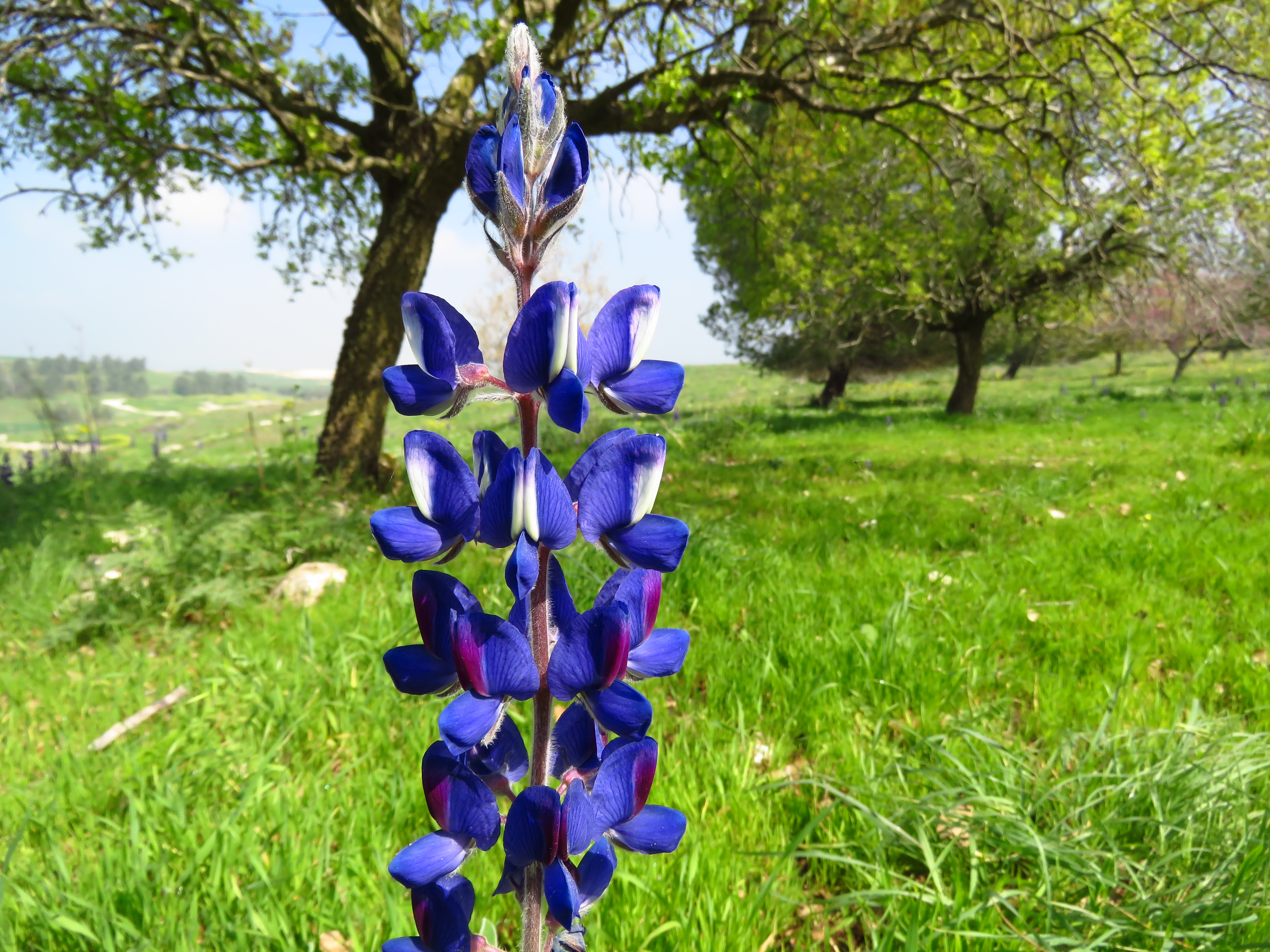

Supplement: Supplementary file 1 [file plants-09-01432-s001.zip › Supplementary for paper/Fig. S4 Flag.JPG]

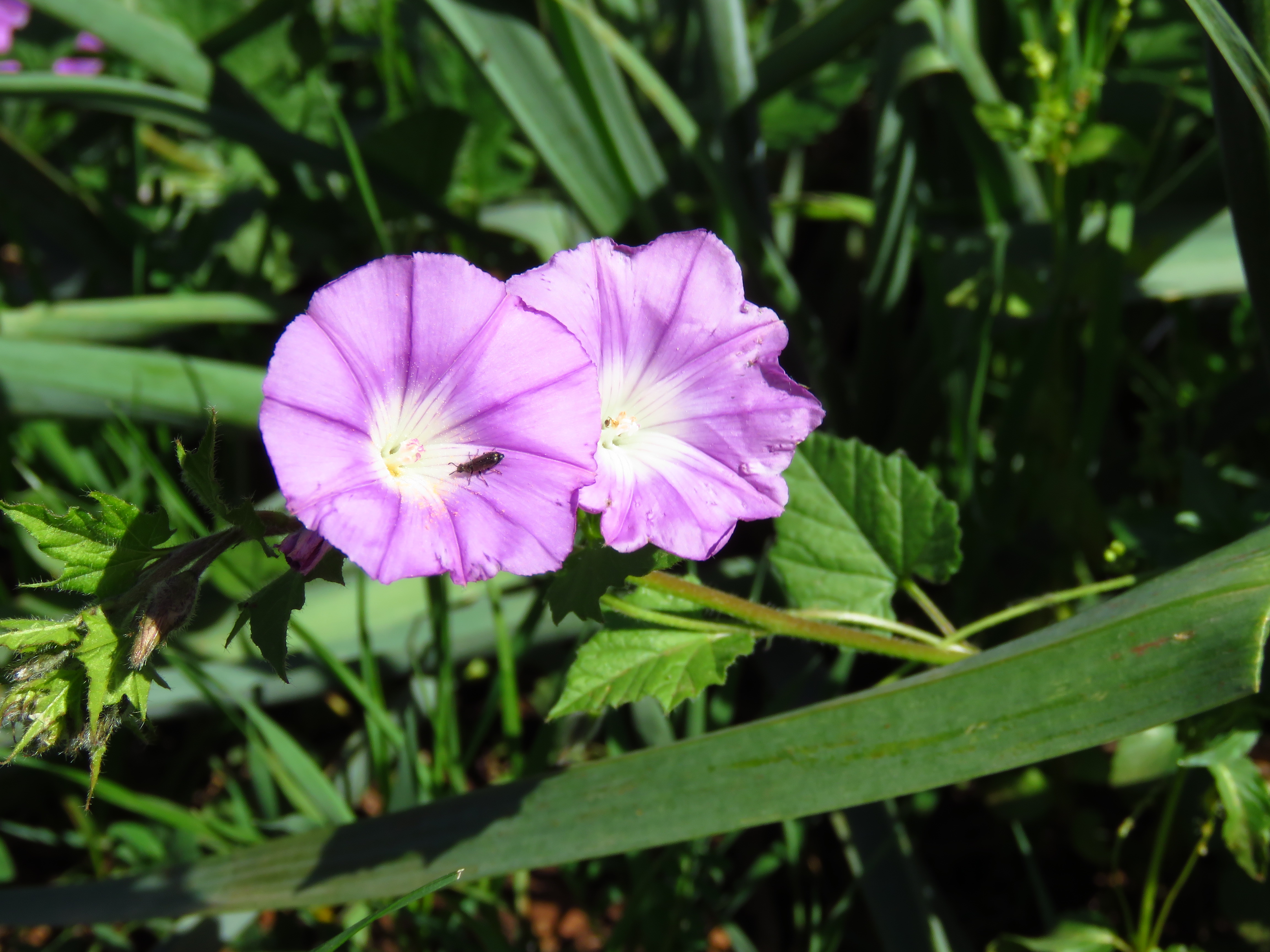

Supplement: Supplementary file 1 [file plants-09-01432-s001.zip › Supplementary for paper/Fig. S5 Funnel.JPG]

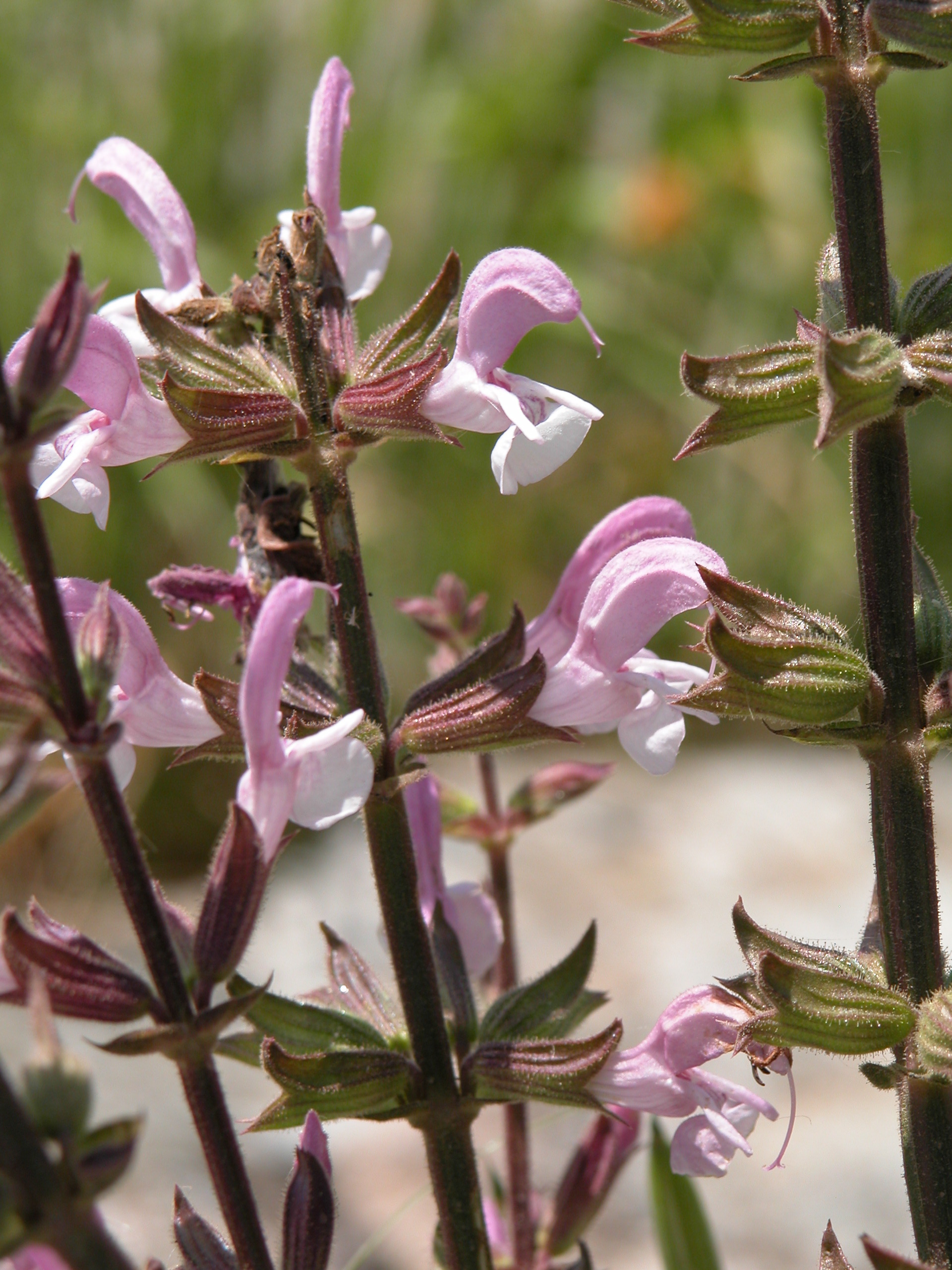

Supplement: Supplementary file 1 [file plants-09-01432-s001.zip › Supplementary for paper/Fig. S6 Gullet.jpg]

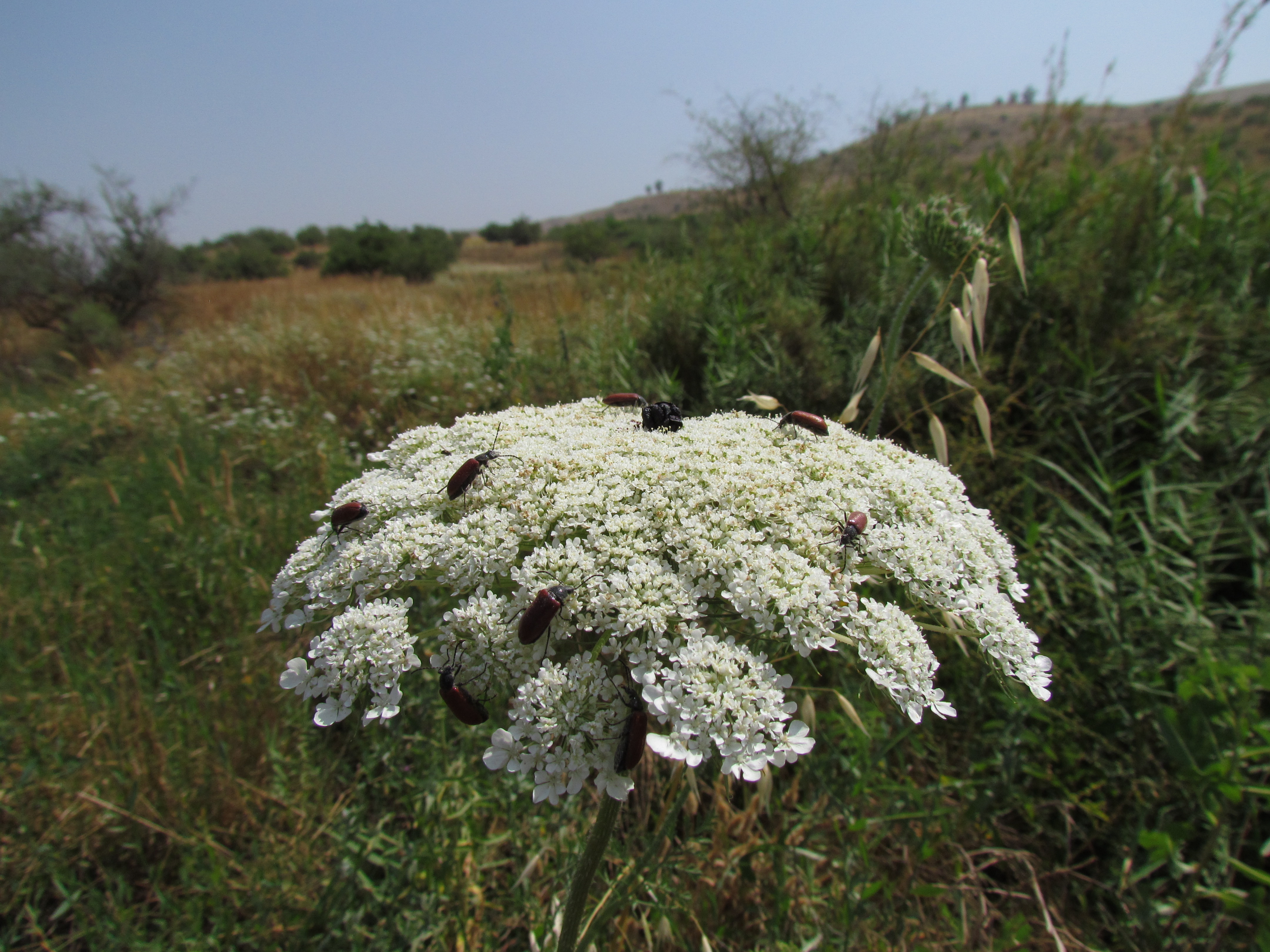

Supplement: Supplementary file 1 [file plants-09-01432-s001.zip › Supplementary for paper/Fig. S7 Head.JPG]
